# Pollinator loss causes rapid adaptive evolution of selfing and dramatically reduces genome‐wide genetic variability

**DOI:** 10.1111/evo.14572

**Published:** 2022-07-25

**Authors:** Jeremiah W. Busch, Sarah Bodbyl‐Roels, Sharif Tusuubira, John K. Kelly

**Affiliations:** ^1^ School of Biological Sciences Washington State University Pullman Washington 99164; ^2^ Trefny Innovative Instruction Center Colorado School of Mines Golden Colorado 80401; ^3^ Department of Ecology and Evolutionary Biology University of Kansas Lawrence Kansas 66045

**Keywords:** Experimental evolution, genetic draft, pollinators, polygenic adaptation, self‐fertilization

## Abstract

Although selfing populations harbor little genetic variation limiting evolutionary potential, the causes are unclear. We experimentally evolved large, replicate populations of *Mimulus guttatus* for nine generations in greenhouses with or without pollinating bees and studied DNA polymorphism in descendants. Populations without bees adapted to produce more selfed seed yet exhibited striking reductions in DNA polymorphism despite large population sizes. Importantly, the genome‐wide pattern of variation cannot be explained by a simple reduction in effective population size, but instead reflects the complicated interaction between selection, linkage, and inbreeding. Simulations demonstrate that the spread of favored alleles at few loci depresses neutral variation genome wide in large populations containing fully selfing lineages. It also generates greater heterogeneity among chromosomes than expected with neutral evolution in small populations. Genome‐wide deviations from neutrality were documented in populations with bees, suggesting widespread influences of background selection. After applying outlier tests to detect loci under selection, two genome regions were found in populations with bees, yet no adaptive loci were otherwise mapped. Large amounts of stochastic change in selfing populations compromise evolutionary potential and undermine outlier tests for selection. This occurs because genetic draft in highly selfing populations makes even the largest changes in allele frequency unremarkable.

With few exceptions, flowering plants do not move as adults, yet reproduction typically involves finding and mating with another individual (i.e., outcrossing; Holsinger 2000; Igic and Kohn 2006). Outcrossing in angiosperms is most commonly achieved by insect pollination, and the question naturally arises how plants respond to an abrupt disruption of this symbiosis (Thomann et al. 2013; Cheptou 2018). In the extreme case of immediate and complete pollinator loss, an obligately outcrossing population will go extinct unless it adapts or colonizes a new environment with pollinators (Gomulkiewicz and Holt 1995; Thomann et al. 2013; Rodger et al. 2021). Given that the pollination symbiosis can be disrupted in many ways, it is not surprising that plants frequently evolve the capacity to self‐fertilize, or mate with themselves (Stebbins 1957; Lande and Schemske 1985). Although transitions from outcrossing to selfing are among the most commonly observed in the flowering plants (Barrett 2002), high selfing rates appear to negatively influence the diversification process (Goldberg et al. 2010); however, the ultimate causes of this macroevolutionary pattern are not well understood (Igic and Busch 2013; Wright et al. 2013; Hartfield et al. 2017).

Naturally selfing populations possess little genetic variation and are highly differentiated from their outcrossing relatives (Schoen and Brown 1991; Ingvarsson 2002; Busch et al. 2011; Goldberg and Igic 2012; Brandvain et al. 2013; Laenen et al. 2018). These patterns are consistent with reductions in effective population size caused by inbreeding (Caballero and Hill 1992; Schoen et al. 1996; Glemin et al. 2006). Selfing increases homozygosity and the coalescence of alleles within individuals is rapidly accelerated (Pollak 1987; Nordborg 2000; Wright et al. 2013). With a higher inbreeding coefficient (*F*), neutral theory predicts that effective population size (*N*_e_) in partially selfing populations at equilibrium will be reduced relative to an idealized Wright Fisher population with *N* individuals ($N_{e}=N/(1+F\;$); Pollak 1987; Caballero and Hill 1992). Perhaps more importantly, inbreeding reduces the effectiveness of recombination (Allard 1975; Kelly 1999) that can greatly amplify background selection and genetic hitch‐hiking (Maynard Smith and Haigh 1974; Barrett et al. 2014; Roze 2016; Hartfield et al. 2017). Selection on a single locus can thus cause rapid (and seemingly stochastic) change at many linked neutral polymorphisms, a phenomenon known as genetic draft (Gillespie 2001; Kelly and Hughes 2019; Buffalo and Coop 2020). Beyond these genetic consequences of selfing, the ecology of self‐fertilization should cause additional declines in *N*_e_ because it fundamentally reduces the number of individuals necessary to make offspring in a population (Baker 1955; Pannell and Barrett 1998; Ingvarsson 2002).

Many studies have compared selfing and outcrossing populations long after their divergence (Glemin et al. 2006; Wang et al. 2021), yet it remains unclear how quickly selfers lose genetic variation and why. Directional selection in highly selfing populations will accelerate the loss of variation beyond the rate expected under genetic drift, because neutral alleles throughout the genome of a selfing lineage will be influenced by natural selection (Robertson 1961; Crow and Denniston 1988; Caballero and Hill 1992; Roze 2016). This influence, essentially genetic draft at the whole genome level, should be enhanced in cases where the initial prevalence of the favorable genotype/phenotype was uncommon (Caballero and Santiago 1995). A narrow spectrum of phenotypes may be selected when pollinators disappear, given that alleles increasing the rate or efficiency of selfing should be rare in historically outcrossing populations (Layman et al. 2017). In addition, the expression of recessive deleterious mutations as homozygotes with selfing further reduces *N*_e_, as the loss of families is typical in studies that have inbred historically outcrossing populations (Willis 1999; Abu Awad and Billiard 2017; Baldwin and Schoen 2019). Substantial reductions in variation, driven by the adaptive evolution of selfing, can constrain the evolutionary potential of lineages soon after they transition away from outcrossing (Haldane 1927; Stebbins 1957; Caballero and Santiago 1995; Glemin and Ronfort 2013; Roze 2016; Abu Awad and Billiard 2017; Hartfield et al. 2017).

In this article, we investigate pollinator loss using experimental evolution. Replicate populations of *Mimulus guttatus*, a predominantly outcrossing species, were maintained with and without access to pollinators for nine generations in a controlled greenhouse setting. After five generations, populations without bees had adapted to the loss of pollinators, exhibiting increased selfed seed production and reduced stigma‐anther distance (Bodbyl‐Roels and Kelly 2011), a key trait determining the ability to self‐fertilize in *Mimulus* (Grossenbacher and Whittall 2011). Experimental evolution was advanced an additional four generations and we here report the results of whole‐genome evolution after nine generations. We use pooled population sequencing to address three questions: (1) To what degree does pollinator loss amplify stochastic allele frequency change, both in terms of the average level of variation (the effective population size effect of selfing) and generate heterogeneity across the genome (a distinct consequence of hitch‐hiking with selfing)? (2) Does selection, combined with inbreeding, produce a distinct stochastic signature from a simple reduction in effective population size? (3) Can we identify loci that were targets of selection, that is, effectors of fitness generated by the mating system treatment? To address these questions, we apply competing evolutionary models to genome‐wide polymorphism data coupled with computer simulations of natural selection with inbreeding.

## Methods

### THE SOURCE POPULATION

**Figure 1:**
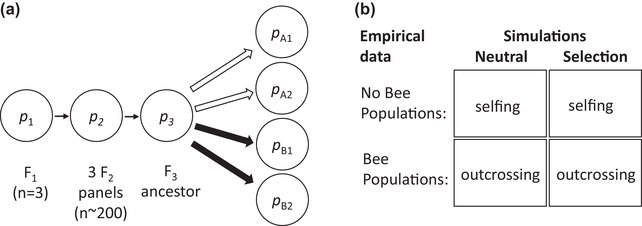
(a) The experimental design. Three F_1_ plants were selfed to generate three F_2_ populations (each consisting of over 200 plants). F_2_ plants were randomly paired (within and across F_2_ populations) and then mated to create a large and outbred ancestor population (F_3_). Populations evolved for nine generations in two treatments: No Bee (unfilled arrows) or Bee (filled arrows). No Bee populations (A1 and A2) reproduced with full selfing, whereas Bee populations (B1 and B2) outcrossed with the aid of pollinating bees. In each population, divergence in allele frequency from the ancestor (e.g., *p*_A1_ – *p*_3_) was independent. (b) To understand the causes of whole‐genome divergence, neutral simulations were compared to those with natural selection. No Bee and Bee populations were informed by simulations with full selfing and outcrossing, respectively.

Plants were ultimately derived from the large Iron Mountain (IM) population of *Mimulus guttatus* (Willis 1993). This population is primarily outcrossing and maintains very high genetic variation, both at the molecular (Puzey et al. 2017) and quantitative trait levels (Willis 1999; Kelly and Arathi 2003). Founders in this experiment were synthesized using descendants of a six‐generation selection experiment on corolla width (Kelly 2008; Fig. 1a). Although this design naturally causes the loss of some standing genetic variation as compared with the IM population, these selection lines retained variation at millions of SNPs throughout the genome (Kelly et al. 2013). From these lines, three unique pairs of small‐ and large‐flowered plants were crossed to generate three F_1_ plants. This design was chosen to capture genetically based variation in floral characters. Each F_1_ was selfed to generate a large F_2_ population, and then F_2_s from each population were randomly paired (within and across the three F_2_ panels) and intercrossed. The resulting (outbred) F_3_ seed collection was the ancestor for all four experimental populations analyzed in this study (Fig. 1a).

### THE GREENHOUSE EXPERIMENT

A diverse array of bees are the primary vectors of pollen at Iron Mountain (Arathi and Kelly 2004) and their exclusion strongly limits seed production (Fishman and Willis 2008). In this experiment, a greenhouse was used to simulate a loss of pollinators (Bodbyl‐Roels and Kelly 2011). The F_3_ seed source was split into two treatments, hereafter referred to as “No Bee” and “Bee,” with two replicate populations in each treatment (No Bee: A1 and A2; Bee: B1 and B2; Fig. 1a). For each population, a fixed seed mass was scattered to soil within each population. The input mass was the same across populations within a generation but adjusted between generations to yield ∼800 adult plants per population. Input seed mass (in grams) for generations 1–9 was 20, 30, 40, 35, 35, 40, 27, 27, and 20, respectively. Soil was watered to simulate field conditions that were moist early with progressive drying. The adult population sizes (number of plants in each population to flower) are reported in Table 1.

**Table 1:** The adult population sizes (*N*) and fitness per adult plant (mg seed produced/individual) reported for generations 1–9 of the selection experiment

Population	A1		A2		B1		B2	
Generation	*N*	Seed Mass/Adult	*N*	Seed Mass/Adult	*N*	Seed Mass/Adult	*N*	Seed Mass/Adult
1	753	1.18	643	1.70	813	6.61	652	9.24
2	424	0.38	458	0.54	652	11.20	488	10.00
3	770	0.54	835	0.41	1353	5.50	816	6.45
4	697	1.90	762	1.38	940	9.54	719	15.76
5	312	8.31	420	4.04	579	9.14	758	13.89
6	1188	0.55	1404	0.11	2268	3.19	2058	2.29
7	953	0.67	788	0.61	1034	4.54	1002	5.45
8	1321	1.37	1388	0.82	1399	12.20	1304	12.48
9	950	2.41	998	1.58	1043	11.45	1023	12.42

During flowering, plants in the B1 and B2 populations were sequentially moved into a separate greenhouse for 2 days, where they received visits from a colony of *Bombus impatiens*. Pollen was cleaned and processed by bees before they were exposed to new plants, preventing gene flow between B1 and B2. The A1 and A2 populations were maintained without pollinators and produced fruits autonomously. Six weeks after planting, all plants were moved to a growth room where they naturally senesced and mature fruits were collected. All seeds produced within each replicate population were bulked prior to initiating the next generation. Plants in the A1 and A2 populations exhibited initially severe declines in per capita seed production in the first few generations of the experiment, although these declines did not severely reduce adult population sizes (Table 1). Per capita seed production rebounded in these populations as they evolved an improved capacity to self‐fertilize over the first five generations (see fig. 2 of Bodbyl‐Roels and Kelly 2011).

### SEQUENCING AND SNP CALLING IN POOLED SAMPLES

After nine generations of selection, an additional generation, comprising 100 plants from each population, was grown for whole‐genome sequencing. Each maternal parent contributed equal seed mass to a bulked seed collection within each of the four descendant populations. DNA was extracted from the seed pool of each population using the Qiagen DNeasy kit and then barcoded sequencing libraries were made using the Illumina DNA Prep kit (previously called the Nextera DNA Flex kit). These libraries were sequenced using Illumina NovaSeq (S4 option) at the University of Kansas Medical center, generating 150‐bp paired‐end reads. Raw reads were trimmed to remove low‐quality bases in TrimGalore version 0.6.6 (Krueger 2015). Reads were aligned to the *M. guttatus* TOL version 5.0 reference genome (Phytozome.net) with default settings in BWA (Li and Durbin 2009). Aligned reads were merged in Samtools version 1.11 and PCR/optical duplicates removed using Picardtools (Broad Institute 2019).

We obtained over 200 million read pairs from each pool (A1: 215M, A2: 227M, B1: 243M, and B2: 205M) and a variant call file was made using VarScan version 2.4.2 (Koboldt et al. 2012). We retained only biallelic SNPs that are also polymorphic in 187 fully genome sequenced lines from IM (Troth et al. 2018). Here, we remapped the sequence data from Troth et al. (2018) to the TOL version 5.0 reference and confirmed that the same alternative bases segregate at homologous positions in each dataset. Insertion‐deletions (indels) were not considered. We removed SNPs if there were fewer than 50 reads in any population or if the total read sum distribution exceeded the 95th percentile of the distribution or if the minor allele frequency was below 5% in all populations. A total of 1,598,153 SNPs remained for downstream analyses. Nucleotide diversity, or the average number of nucleotide mismatches per site (π), was calculated in each population in 50‐kb windows across each chromosome.

### ESTIMATING THE MAGNITUDE OF NEUTRAL EVOLUTION IN EACH POPULATION

In this experiment, evolutionary change within populations reflects the combined action of genetic drift and natural selection, both at polymorphisms affecting fitness and linked SNPs. If most SNPs were neutral with respect to fitness over the course of the experiment, we can use the overall distribution of allele frequency changes to estimate the magnitude of change at neutral SNPs. Here, the angular (arcsin square root) transformation was applied to frequencies: $x=2\mathrm{si}n^{-1}\sqrt{p}$, where *p* is the untransformed allele frequency, and *x* is measured in radians (Fisher and Ford 1947). Assuming a Wright‐Fisher population model, the (short term) change in *x* over *t* generations will be normally distributed with mean zero and variance = $\frac{t}{2N_{e}}+\frac{1}{2n}+\frac{1}{m}$, where *N*_e_ is the effective population size, *n* is the number of plants bulked for sequencing, and *m* is read depth at a SNP. The first two terms in this variance are the same for all SNPs in the genome, whereas the last will differ owing to varying read depth across SNPs. We use the procedures outlined in Kelly et al. (2013) to estimate the “null variance” (*v*) within each population. The procedure obtains a robust estimate for the variance of observed divergence, unaffected by outlier loci under selection. We then subtract off the variance owing to finite sequencing depths. The null variance within a population represents the aggregate of allele frequency dispersion, which includes genetic drift and draft plus the additional round of sampling with the bulking of DNA molecules in each sequencing pool.

Because we do not have the ancestral population, $v_{A1},\;v_{A2}$, $v_{B1},\;\mathrm{and}\;v_{B2}$ are estimated from the divergence between populations (e.g., the variance in null divergence between A1 and A2 is $v_{A1}+v_{A2}$). We used generalized least squares to distill the six distinct pairwise contrasts between populations into the four null variances. The model is $y=X\hat{\beta }$ + error, where *y* is the vector of population pair divergences, $\hat{\beta }$ is the vector of null variances, and *X* is a matrix of indicator variables relating populations to the pairwise comparisons. The variance‐covariance matrix of null divergences (*V*) was generated by splitting the whole‐genome dataset into windows of 500 SNPs, bootstrapping the SNP dataset 1000 times, and estimating the six null divergences in each replicate. The vector of null variance parameters was then estimated as $\hat{\beta }={\left(X^{T}V^{-1}X\right)}^{-1}\;XV^{-1}y$ (Lynch and Walsh 1998, p. 843). We first applied this procedure to obtain genome‐wide estimates for $v_{A1},\;v_{A2}$, $v_{B1},\;\mathrm{and}\;v_{B2}$ and then separately to each chromosome to estimate intragenomic variability in divergence. These statistics characterize divergence and are thus results of the experiment. However, we also used the null variance estimates to calibrate the null model when later testing for divergence driven by selection. We estimated pairwise *F*_ST_ between populations, which is a measure of allele frequency variance among populations standardized by the total variance. *F*_ST_ among pairs of populations was calculated using an ANOVA framework (Weir and Cockerham 1984), implemented in the poolfstat R package (Hivert et al. 2018).

### SIMULATIONS OF GENOME EVOLUTION WITH LINKAGE

To understand the causes of divergence at the level of whole genomes in the experiment, we conducted individual‐based simulations of evolution (Fig 1b). Our goal involved comparing neutral simulations to those with natural selection to test the prediction that the whole‐genome divergence and its variability among chromosomes implicated selection. To inform outcomes of evolution in the Bee and No Bee populations, we simulated populations that reproduced through random outcrossing and full selfing, respectively (Fig. 1b). In all simulations, whole chromosomes of individuals were tracked. To initialize these populations, the diploid genotypes of *N* founders were randomly drawn using the average frequency of alleles at each SNP in the four experimental populations. After founding, the population experienced nine generations of evolution, where *N* remained constant. During the production of random gametes by parents, a single recombination breakpoint occurred at a random internal position of a chromosome, consistent with inferred patterns of recombination in *Mimulus guttatus* (Flagel et al. 2019). Two thousand SNPs per chromosome were chosen for simulations to speed calculations, which were conducted in Python version 3.0.

In the neutral simulations, *N* random seed parents were chosen to reproduce. Pollen parents were randomly drawn from the population in outcrossing simulations or were identical to seed parents in selfing simulations. We ran these simulations over a broad range of *N* values to identify the specific value of *N* that produced results that most closely fit the observed null variances in each experimental population. Once those *N* values were identified, we compared the frequency of minor alleles in each experimental population to the distribution in the corresponding neutral simulations. We generated 1000 simulations of neutral evolution and chose the 10 simulations with null variances closest to that observed in each experimental population. The probability densities of minor alleles in these simulations and the experimental population were then compared to evaluate the degree to which the empirical data support the hypothesis of neutral evolution.

To model natural selection, the simulations were otherwise identical except for the inclusion of mutations that influence fitness. These simulations included a rare mutation that increased fitness at one or two loci to demonstrate the influence of natural selection on the amount of stochastic change in genomes. In these cases, parents were drawn randomly with a probability equal to their relative fitness. Models of strong selection on initially rare mutations were chosen because they predict a rapid increase in fitness over the first five generations of evolution, with subsequent attenuation of the evolutionary responses (Table 1). In these models, the locus harboring a favored mutation was positioned in the middle of a chromosome. In the two‐locus model, loci were in the middle of different chromosomes and individual fitness equaled the multiplicative fitness of genotypes across loci. In all simulations with selection, runs required that the favored alleles at each locus reached a frequency exceeding 0.50. To compare the outcomes of neutral models to models with selection, a total of 1000 independent evolutionary replicates were generated in each case. To evaluate the fit of each model to the observed data, we compared the mean null variance and the coefficient of variation across chromosomes in the null variance to the simulations.

### TESTS OF ADAPTATION USING THE SNP OUTLIER FRAMEWORK

After establishing the impact of treatments on whole‐genome divergence, we examined their implications on the ability to infer SNPs under selection. We here elaborate the likelihood‐based approach of Monnahan and Kelly (2017) and Kelly and Hughes (2019) to determine the best fitting evolutionary model at each SNP. For each SNP, the observed allele frequencies in all populations ($x_{a1},x_{a2},$ etc.), null variances ($v_{a1},v_{a2},$ etc.), and SNP‐specific read depth variances ($1/m_{a1},\;1/m_{a2}$, etc.) were used to calculate the likelihood of competing models (Table 2). The null model (model 0) was genetic drift, with a single allele frequency parameter. Four models had an additional parameter denoting adaptation in one population (models 1–4). Parallel adaptation (model 5), or similarity by treatment, had a shared allele frequency for the No Bee populations and a shared allele frequency for the Bee populations. More complex models considered parallelism within the No Bee populations (model 6), parallelism within the Bee populations (model 7), or independence in all four populations (model 8). Maximum likelihood estimates of parameters in each model were inferred using the optimize.fmin_l_bfgs_b function in SciPy (Virtanen et al. 2020). A log likelihood (LL) for each model was computed by adding the Ln(likelihood) values across populations. Model support was calculated using AIC=2k−2LL, with *k* denoting the number of estimated allele frequencies (Table 2).

**Table 2:** Competing models of SNP evolution. Models 1–4 allow adaptation in a single population. Models 5–7 allow various forms of parallel adaptation to treatments. Each of the *k* allele frequency parameters were estimated with maximum likelihood

Model	Process	Model Parameters	*k*
0	Random drift	*p*_0_: all populations	1
1	A1 adaptation	*p*_0_: A2, B1, B2	2
		*p*_1_: A1	
2	A2 adaptation	*p*_0_: A1, B1, B2	2
		*p*_1_: A2	
3	B1 adaptation	*p*_0_: A1, A2, B2	2
		*p*_1_: B1	
4	B2 adaptation	*p*_0_: A1, A2, B1	2
		*p*_1_: B2	
5	Parallel adaptation	*p*_0_: A1, A2	2
		*p*_1_: B1, B2	
6	No Bee parallelism	*p*_0_: A1, A2	3
		*p*_1_: B1	
		*p*_2_: B2	
7	Bee parallelism	*p*_0_: B1, B2	3
		*p*_1_: A1	
		*p*_2_: A2	
8	Independence	*p*_0_: A1	4
		*p*_1_: A2	
		*p*_2_: B1	
		*p*_3_: B2	

To test hypotheses of selection, models were compared to the null model. For SNPs where drift was not the best fitting model by AIC, likelihood ratio tests (LRTs) of the difference between the best fitting model's log likelihood (e.g., LL_1_) and the null likelihood (LL_0_) were computed from 2(LL_1_ – LL_0_). LRT statistics are approximately *χ* ^2^ distributed with degrees of freedom equal to the *k*_1_ – *k*_0_. To account for multiple comparisons, the threshold of significance was determined using Sidak's correction with a false discovery rate (FDR) of 0.05 (Sidak 1967). Manhattan plots displaying the locations of SNPs exceeding this threshold were generated in qqman (Turner 2018). To further inform tests of selection, we classified SNPs within genes, first as within exons or introns, and for the former as synonymous or nonsynonymous. These categorizations were extracted from annotations in the *M. guttatus* reference genome using SnpEff (Cingolani et al. 2012). All SNPs nearby indels (±5 bp) were also identified by comparing their location with indels identified in Samtools version 1.11.

## Results

### WHOLE‐GENOME RESPONSES TO THE EXPERIMENTAL TREATMENTS

**Figure 2:**
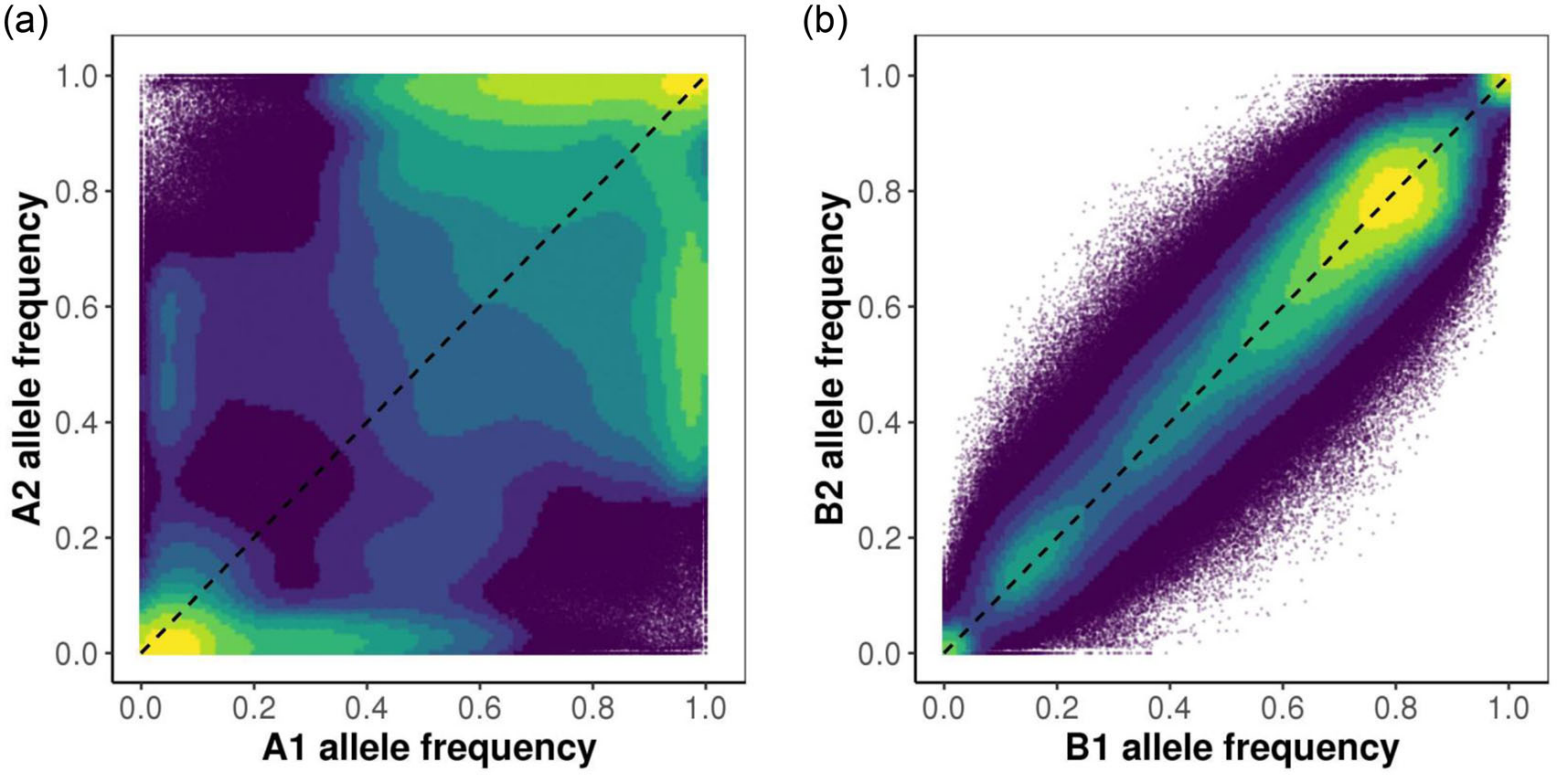
Divergence in allele frequency between populations within the same experimental treatment. (a) No Bee populations (A1, A2) that rely on self‐fertilization. (b) Bee populations (B1, B2) that were visited by pollinating bees. Points are colored by deciles of kernel density, with warmer colors denoting regions of higher SNP density. The dashed lines denote expectations prior to experimental evolution.

No Bee populations exhibited greatly elevated genetic differentiation compared to Bee populations (Fig. 2). *F*_ST_ values between each pair of populations ranged from 0.0207 to 0.2814 (A1 vs. A2 = 0.2814; A1 vs. B1 = 0.1079; A1 vs. B2 = 0.1131; A2 vs. B1 = 0.2299; A2 vs. B2 = 0.2357; and B1 vs. B2 = 0.0207). Lineage specific null variances were 0.0138 for B1, 0.0235 for B2, 0.2554 for A1, and 0.8545 for A2. The higher values for A1 and A2 indicate greatly elevated allele frequency change in the No Bee treatment (Fig. 2). The square‐root of the null variances indicates the typical change in allele frequency in each population. In A1, a SNP with initial *p* = 0.5 exhibits an average Δp of 0.24 (the final allele frequency in the pool would be 0.26 or 0.74). This average change is even larger for A2 (Δp≈0.40), but much smaller in B1 (Δp≈0.06) and B2 (Δp≈0.08).

The much greater divergence of the No Bee populations cannot be explained by differences in adult population sizes. Hundreds of individuals were grown each generation and mean population sizes were only slightly lower in the No Bee populations (A1 = 818.67; A2 = 855.11) relative to Bee populations (B1 = 1120.11; B2 = 980; Table 1). In line with greater stochastic evolution without pollinators, No Bee populations exhibited less nucleotide diversity (A1 π = 0.02245, A2 π = 0.01952) compared to Bee populations (B1 π = 0.02569, B2 π = 0.02582), a pattern that was observed across all chromosomes (Fig. S1).

### USING SIMULATIONS TO UNDERSTAND THE CAUSES OF GENOME‐WIDE PATTERNS

**Figure 3:**
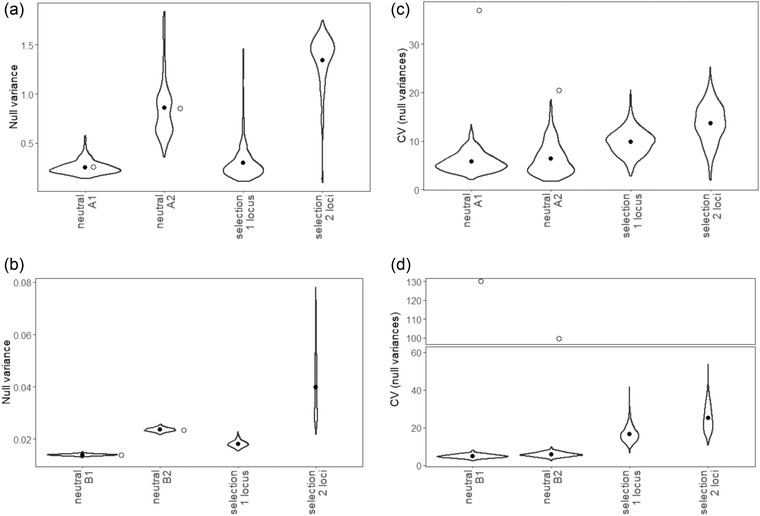
Comparing simulation results to empirical data in the experiment. (a, b) Distributions of the genome‐wide null variance, and (c, d) interchromosomal variance in the null variance (measured by the coefficient of variation) in 1000 independent simulations. The top row considers selfing (A1 and A2 populations), whereas the bottom row considers outcrossing (B1 and B2 populations). Filled circles denote means in simulations, whereas open circles denote the observed values of statistics in the experiment. The neutral simulations used *N* values that best reproduced observed null variances. We set *N* = 500 for the selection simulations, a large value approximating observed adult population sizes (Table 1), with selection coefficient *s* = 0.8, and initially rare, favored alleles (*p* = 0.02). Note the *y*‐axis break in panel (d).

By considering a range of *N* values, we found the size for each population that, with strictly neutral evolution, yields an average null variance that matches the observed null variances: *N*[A1] = 43, *N*[A2] = 13, *N*[B1] = 366, and *N*[B2] = 219. The much smaller *N* for A1 and A2 is consistent with greater stochastic changes in allele frequency within the No Bee populations. Simulations of selfing populations with *N* < 50 exhibit large variation in the estimated null variance among replicates (Fig. S2). In fact, neutral simulations with selfing and *N* = 30 routinely yield null variances as low as A1 (0.255) and as high as A2 (0.855; Fig. S2). Although adjusting *N* for each population allows us to match the genome‐wide null variances observed in populations (Fig. 3a,b), the neutral simulation does not reiterate a second key feature of the data—the amount that sequence divergence varies among chromosomes (Fig. 3c,d). We expect that natural selection will inflate the variance among chromosomes, with greater change occurring on chromosomes enriched for loci under selection. In the No Bee populations, the coefficient of variation (CV) for chromosome‐specific null variances is four‐ to eightfold greater than predicted by the calibrated neutral model (Fig. 3c). In the Bee populations, the inflation is even more extreme (over 20×; Fig. 3d).

We consider simulations with selection motivated by three features of the results: (1) through the first five generations, there was clear evidence of adaptive evolution of quantitative traits and of mean fitness in No Bee populations (Bodbyl‐Roels and Kelly 2011); (2) although the neutral simulation can reiterate the high null variances of A1 and A2, they require population sizes ≈20× lower than observed numbers of flowering plants in these populations (Table 1); and (3) the high interchromosomal variation in divergence is indicative of selection.

We first considered a single locus with the favored allele initially rare (third section of each panel in Fig. 3). A model with an initially rare mutation under strong selection (*s* = 0.8) was chosen because its spread emulates the rapid increase in self seed production in No Bee populations followed by little change after five generations of evolution (Table 1). This evolutionary scenario generates a broad distribution of genome‐wide null variances that overlap the observed values from populations A1 and A2 (Fig. 3a). Selection on two loci under strong selection (*s* = 0.8) causes substantially higher null variance, as expected with additional linked selection in the genome. In large outcrossing populations (*N* = 500), selection on one locus produces outcomes in between those in smaller populations (*N* = 219 or 366, the best fitting parameters in B2 and B1) without natural selection. Strong selection on two loci causes higher null variance, consistent with selection causing stochastic change at linked loci (Fig. 3a,b). The mean null variance and its range are much larger in fully selfing populations (Fig. 3a) compared to those that are outcrossing (Fig. 3b).

Regardless of the mating system of populations, the average variability in stochastic changes increases with each additional locus under selection. This measure of variability exhibited broader distributions in outcrossing populations, along with a greater tendency to increase with the number of selected loci (Fig. 3c,d). Variability in the two‐locus models with selfing overlapped the value observed in the A2 population (CV = 20.41), whereas all selfing models fell below that observed in the A1 population (CV = 36.97). This departure implies a slightly more complex genetic basis of adaptation in the A1 population compared to the A2 population (Fig. 3c). In contrast, variability in all outcrossing models fell markedly below the values observed in the B2 population (99.72) and the B1 population (130.03). Subtle effects of selection at many loci in the Bee populations are therefore implicated, consistent with their markedly nonneutral allele frequency distributions (AFDs) (Fig. 3d).

**Figure 4:**
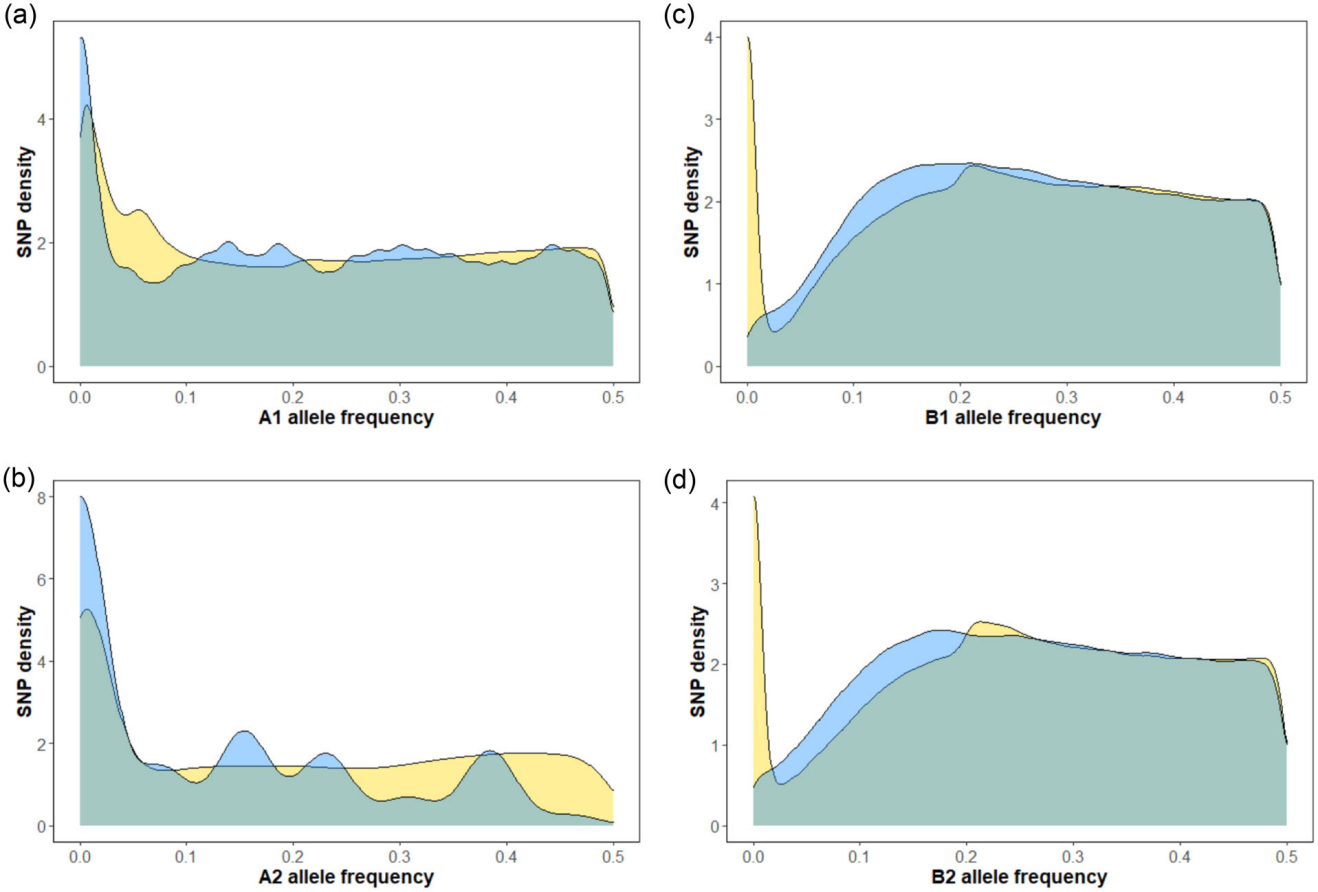
The distribution of minor allele frequencies in each population after nine generations of evolution. (a) A1 population; (b) A2 population; (c) B1 population; and (d) B2 population. Golden and blue denote the empirical distributions and those generated in neutral simulations, respectively. The population size in each neutral simulation equaled the *N* value producing the closest fit to the empirical null variance. Results of 10 independent simulations are shown, which assumed no selection and linkage among 2000 SNPs per chromosome.

Neutral simulations using the best‐fitting number of individuals (*N*) in the No Bee populations produce AFDs that generally resemble empirical distributions (Fig. 4a,b). In both of the No Bee populations, fewer rare variants are evident in comparison to the neutral simulations (Fig. 4a,b). Such a pattern would arise if selection of several competing, fully selfing lineages in the No Bee populations prevented the loss or fixation of neutral variants trapped on their genetic backgrounds (Fig. 4a,b). In contrast, a clear excess of rare variants was evident in Bee populations compared to the neutral simulations, consistent with purifying selection reducing the frequency of harmful alleles throughout the genome (Fig. 4c,d). Departures between the empirical and simulated AFDs are generally consistent with genome‐wide influences of natural selection, especially in Bee populations, where recombination was effective given the outcrossing mode of reproduction.

### TESTS OF ADAPTATION USING THE SNP OUTLIER FRAMEWORK

In considering SNPs in the evolved populations, random genetic drift was the most supported model when considering the minimum AIC criterion (57.85% of all SNPs). The simplest nonneutral models received similar levels of support at this broad level (model 1: A1 adaptation = 6.14%; model 2: A2 adaptation = 6.90%; model 3: B1 adaptation = 9.56%; model 4: B2 adaptation = 9.93%; model 5: parallel adaptation = 8.71%). More complex models of adaptation received little support across SNPs (models 6–8: total support = 0.90%). These patterns (fraction of SNPs favoring each model) are generally reiterated by simulations with purely neutral evolution, at least if we use *N*_e_ values tuned to match the observed null variances. Across 10 whole genome simulations, random genetic drift was the most supported model when considering the minimum AIC criterion (58.89% of all SNPs). The simplest nonneutral models received similar levels of support at this broad level (model 1: A1 adaptation = 7.93%; model 2: A2 adaptation = 6.14%; model 3: B1 adaptation = 8.11%; model 4: B2 adaptation = 9.10%; model 5: parallel adaptation = 8.34%). More complex models of adaptation received little support in the neutral simulations (models 6–8: total support = 1.47%), also mirroring the real data.

**Figure 5:**
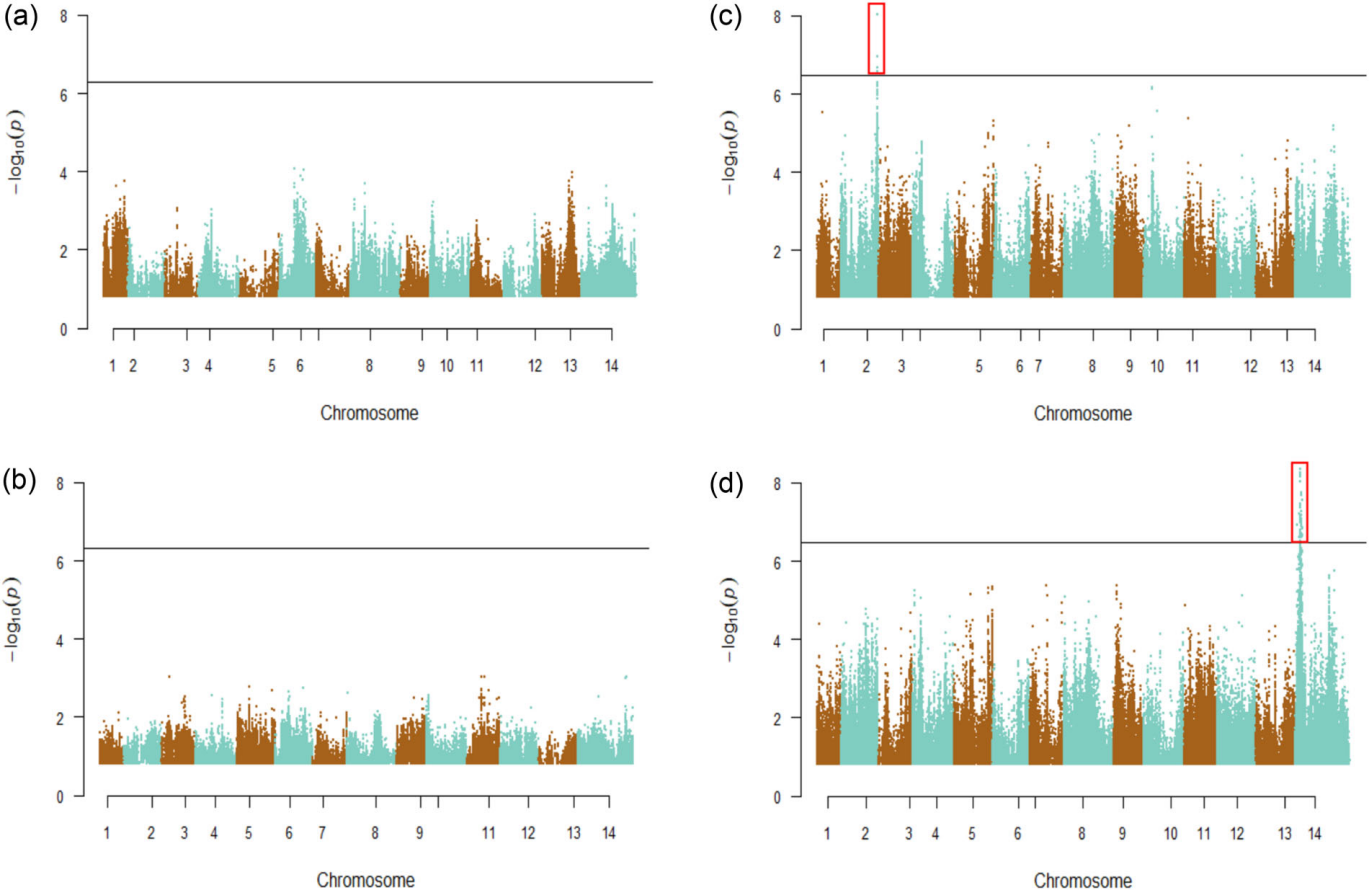
Outlier tests of adaptation in a single population. (a) A1; (b) A2; (c) B1; and (d) B2. *P*‐values were generated from likelihood ratio tests of best fitting models against random drift (model 0). Red boxes denote outlier SNPs, with horizontal lines depicting the threshold *P*‐values with genome‐wide FDR = 0.05.

Slight differences in the log‐likelihood of different models are sufficient for AIC to suggest selection, but large differences are required for an LRT to reject drift in favor of a particular selection model at genome‐wide significance levels. No SNPs were close to this threshold for adaptation in the No Bee treatments (Fig 5a,b). In contrast, we identified two regions of the genome indicating adaptation in the Bee populations (Fig. 5c,d). Four SNPs on chromosome 2 supported the model of adaptation in the B1 population (Fig. 5c), and 41 closely linked SNPs on chromosome 14 supported the model of adaptation in the B2 population (Fig. 5d). An additional two SNPs supported independence on chromosome 1. Most selected SNPs were noncoding and nearby genes that serve a diversity of functions (Table S1).

Differences in statistical power are a key issue for selection tests within the Bee and No Bee populations. The high level of genome‐wide stochastic change in the No Bee populations makes it essentially impossible to reject the drift model. Models 1 and 2 simply cannot produce likelihoods that are sufficiently large relative to model 0 so that *P*‐values are low enough to pass the threshold (Fig. S3). The strongest possible signal for population‐specific adaptation is that a rare allele increases to fixation within the adapting population but remains rare in the other populations. In the A2 population, an initially rare allele (inferred by a final frequency <0.1 in all other populations) went to fixation at 22 distinct SNPs. None of these tests approached genome‐wide significance for A2 adaptation (Fig. S3). In contrast, much less stochastic change (i.e., smaller null variances) in the Bee populations allows LRTs to reject neutrality at least in cases where natural selection is sufficiently strong (Fig. S3).

## Discussion

### POLLINATOR LOSS AND THE EVOLUTION OF SELFING

Reductions in genetic variation within selfing populations relative to closely related outcrossers are commonplace, typically exceeding expectations based on neutral models (Busch et al. 2011; Pettengill and Moeller 2012; Brandvain et al. 2013; Arunkumar et al. 2015; Laenen et al. 2018). Because most of these studies compare populations that diverged many thousands of generations in the past, it has been challenging to infer how quickly selfing reduces variation and why. In this study, shifts to selfing caused by an experimental loss of insect pollinators caused phenotypic adaptation, large changes in allele frequencies, and declines in genome‐wide variability on a rapid timescale (Bodbyl‐Roels and Kelly 2011). Specifically, No Bee populations exhibited greatly elevated stochastic changes in allele frequency and 13%–24% reductions in nucleotide diversity after nine generations of experimental evolution. To understand the likely reasons why these empirical patterns were generated in the experiment, we simulated the evolution of populations under neutrality and in cases where favored alleles spread at a small number of loci.

### SELECTION AND WHOLE‐GENOME RESPONSES TO THE EXPERIMENTAL TREATMENTS

Adaptation in the No Bee populations was implicated as a primary driver of the loss of variation. The harmonic mean numbers of adults were 13%–30% lower in populations without bees, consistent with the expression of genetic load in historically outcrossing populations that abruptly transition to selfing (Willis 1999; Abu Awad and Billiard 2017; Baldwin and Schoen 2019). However, incrementally lower population sizes in No Bee populations are insufficient to explain very large increases in random allele frequency change within these populations. Variance in adult reproductive success can reduce *N*_e_ relative to the census population size (Crow and Denniston 1988). Fishman and Willis (2008) compared seed production of natural plants visited by pollinators to that of caged plants (insects excluded). Natural seed production declined with pollinator exclusion (μ_natural_ = 32.96 seeds/plant vs. μ_caged_ = 5.93 seeds/plant), and the coefficient of variation in individual seed number was 65% higher in caged plants (CV_natural_ = 1.10 vs. CV_caged_ = 1.82). Bodbyl‐Roels and Kelly (2011) found considerable heritability of selfed seed set in these populations after five generations of selection (*h* ^2^ = 0.20–0.40), consistent with a role for selection on the genetic component to generate correlated allele frequency changes across generations, which will greatly amplify the cumulative change (Robertson 1961; Buffalo and Coop 2020).

We considered a specific genetic model for increased selfing in our simulations (Fig. 3). High fitness without pollinators was conferred by initially rare genotypes at one or two loci. These simulations matched the observed results of the experiment much better than neutral simulations. For one, the selection simulations yielded the high null variances observed in the No Bee populations even with large population sizes. In contrast, neutral simulations require population sizes at least an order of magnitude lower than those observed in the experiments (Table 1). Even with small population sizes, the neutral simulations cannot reiterate the empirical variability among chromosomes in the null variance (Fig. 3c,d). In other words, the interaction of selection and selfing generates patterns that cannot be accommodated by a simple adjustment to the overall effective population size (*N*_e_). Selection on one or two loci with inbreeding elevates the variance among chromosomes, although not to the level observed in population A1 (Fig 3c). In outcrossing simulations, selection also inflates the interchromosome variance, although not nearly to the level observed in the B1 and B2 populations (Fig 3d).

The imperfect prediction of interchromosomal differences in divergence may indicate that adaptation was generated by a polygenic response, particularly in the Bee populations. Indeed, as described in detail in the next section, we could not identify loci under selection in the No Bee populations and thus cannot empirically justify a one or two‐locus model. However, the most important effect of selection may not depend strongly on the number of loci. A population that loses pollinators and is compelled to reproduce entirely by selfing is a collection of reproductively isolated yet competing lineages (Heller and Maynard Smith 1979; Pamilo et al. 1987). If one or a few of these lineages rapidly increase via selection, it/they will exclude the entire genome of other lineages. The favored lineage may succeed because it became homozygous for a favorable mutation at one locus or happened to have favorable alleles at numerous loci. Regardless, after the lineage expands, the population will exhibit a genomic polymorphism signature resembling recovery from a severe bottleneck, even though selection and not demography was the ultimate cause (Caballero and Santiago 1995).

In comparative studies, the arrow of causation between selection of selfing and reduced effective population size is rarely straightforward (Barrett et al. 2014; Koski et al. 2019). Here, the pollinator environment was experimentally controlled. Populations that lost pollinators rapidly evolved flowers with a reduced stigma‐anther distance and simultaneously adapted to produce more selfed seed (Table 1; Bodbyl‐Roels and Kelly 2011). Adaptive evolutionary transitions from outcrossing to selfing occur frequently in nature, committing selfers to elevated rates of extinction and speciation (Goldberg et al. 2010). Whether the loss of adaptive potential in selfing lineages coincides with their transition from the outcrossing condition fundamentally depends on the strength of selection and the influence of the mating system on recombination (Maynard Smith and Haigh 1974). Although many flowering plants are heavily dependent on pollinators to produce seed (Rodger et al. 2021), selfing mutations have extremely varied effects on whether plants primarily outcross or self‐fertilize (Piper et al. 1984; Stone et al. 2014; Layman et al. 2017). Mutations that are selected yet do not commit individuals to high selfing rates will displace alleles that enforce outcrossing, but the selective sweep would not necessarily translate into declines in variability across a chromosome (Barrett et al. 2014; Herman and Schoen 2016). In contrast, mutations that spread in a fully selfing context will reduce variability across the whole genome as they sweep to high frequencies (Maynard Smith and Haigh 1974; Caballero and Santiago 1995).

The influence of selection on linked, neutral SNPs genome wide has received increased attention (Gillespie 2001; Kelly and Hughes 2019; Buffalo and Coop 2020). Natural selection not only depresses variation at SNPs that influence fitness, but these effects extend to neutral SNPs in close linkage (Maynard Smith and Haigh 1974; Begun and Aquadro 1992). Perhaps more importantly for the present study, linked selection, or genetic draft, may be as or more important than classical genetic drift in experimental evolution studies (e.g., Kelly and Hughes 2019). The excess of rare alleles in the Bee populations (Fig. 4c,d) and amplified variation among chromosomes in the degree of random changes (Fig. 3d) suggest subtle influences of selection on many genetic variants throughout the genome. Although infrequent hitchhiking events contribute to departures from neutrality (Maynard‐Smith and Haigh 1974), background selection can much more readily depress levels of variation at closely linked yet neutral SNPs throughout the genome (Cvijovic et al. 2018). Although theoretical predictions on the influence of background selection consider populations near equilibrium (Charlesworth et al. 1993; Roze 2016), the large excess of rare variants in Bee populations in this experiment suggests genomes may demonstrably respond to this process in nine or fewer generations (Fig. 4c,d).

The initiation of populations in this study involved very few founders (Fig. 1) that likely enhanced the signal of background selection at the level of whole genomes. Plants descended not directly from the IM population but instead from an artificial selection experiment that favored small or large flowers, respectively (Kelly et al. 2013). Although millions of IM SNPs remain polymorphic in these selection lines (Kelly et al. 2013), only three distinct pairs of plants from the small and large lines were intercrossed to start material for this experiment (Bodbyl‐Roels and Kelly 2011). Even though much of the ancestral variation from IM would therefore be lost, harmful and historically rare mutants that survive this population bottleneck would exceed their low equilibrium frequencies under mutation‐selection balance (Kirkpatrick and Jarne 2000). At the onset of experimental evolution, such mutations would depress fitness in the homozygous state (Willis 1999). Purifying selection on a temporarily elevated load of deleterious mutations is predicted to cause an excess of rare mutations in large, outcrossing populations with effective recombination (Cvijovic et al. 2018). Such a signature attenuates in selfing populations, however, because lineages driven to high frequency by selection bring along harmful mutations trapped in their genetic background (Heller and Maynard Smith 1979; Pamilo et al. 1987). In fully selfing populations, major constraints on recombination limit the decoupling of mutations that either increase or decrease fitness in a lineage (McDonald et al. 2016).

### TESTS OF ADAPTATION USING THE SNP OUTLIER FRAMEWORK

Beyond these patterns realized at the level of whole genomes, tests of outlier SNPs found evidence of natural selection promoting divergence in two populations. Four selected SNPs on chromosome 2 were identified in the B1 population, and a collection of 41 SNPs on chromosome 14 were identified in the B2 population. In the latter case, most mutations are found in noncoding regions, although four silent and three intron SNP outliers reside within this large 2.25 Mbp window. All but one of these SNPs lack genetic variation in the B2 population but segregate at intermediate frequencies elsewhere, with an average minor allele frequency ranging between 0.31 and 0.46. Given that genetic drift was somewhat limited in scope in the B1 and B2 populations, SNPs with this level of differentiation stand out from the background of whole‐genome changes, implying an additional process promoting their divergence over neutral expectations. Although it is not possible to determine the putative impacts of these SNPs on fitness, the clustering of differentiated SNPs is consistent with the action of linked selection (Kelly and Hughes 2019). Beyond these two independent instances of adaptation in each of the Bee populations, few other outlier SNPs were inferred in this experiment (Table S1).

Although polygenic adaptation will have slight effects on selected SNPs (Berg and Coop 2014; Buffalo and Coop 2020), searches for the footprints of strong natural selection in genomes rely upon distinctions between those SNPs and the rest of the genome. Such an “evolve‐and‐resequence” approach can identify targets of selection when these loci become highly differentiated relative to the remainder of the genome (Lewontin and Krakauer 1973; Nuzhdin and Turner 2013; Huang et al. 2014). A second signal is that selected SNPs should show parallel change in replicate populations with the same treatment (fitness regime), whereas neutral SNPs should not (Vlachos et al. 2019). Our results show that selfing can undermine tests based on both signals. First, when genetic drift alone is strong enough to produce highly differentiated SNPs, SNPs under selection will not “stand out” from the background of random changes (Grueber et al. 2013; Kelly et al. 2013). Second, amplified stochasticity can make the response of selected loci idiosyncratic to each replicated population. A parallel response to selection is impossible if an allele that increases in one replicate population is lost in another due to drift or draft. This is most likely when favorable alleles are initially rare and thus vulnerable to loss in the early stages of adaptation (Gillespie 2001).

Adaptive evolution of selfing is among the most common evolutionary transitions yet this reproductive mode is often synonymous with low genetic variation (Glemin et al. 2006; Wright et al. 2013). This experiment shows that adaptive transitions to selfing have the potential to rob populations of genome‐wide variation necessary for adaptation to future environmental challenges (Goldberg et al. 2010). At least in extreme cases of complete pollinator loss, identifying the genomic targets of adaptation in selfing lineages will be challenging using polymorphisms alone (Slotte et al. 2012), and prospects for parallel adaptation from standing variation seem similarly remote using the evolve‐and‐resequence approach. Beyond the genetic consequences of selfing, this study joins others in demonstrating inferential weaknesses of the SNP outlier framework in highly inbred or bottlenecked populations (Foll and Gaggiotti 2008; Bollmer et al. 2011; Grueber et al. 2013; Leigh et al. 2021). It is important to note that the pooled sequencing approach generally weakens the power of selection tests compared to cases where haplotype‐level information is included (Kessner et al. 2013). Regardless of whether tests are applied to single SNPs or haplotypes, the search for sites under selection should be compromised whenever genetic draft in highly selfing populations causes even the largest possible changes in allele frequency to be mundane (Navascues et al. 2021).

## Conclusions

This experiment represents a case study of evolutionary rescue, where pollinator loss represents an abrupt environmental change that drastically reduced per capita fitness (Bodbyl‐Roels and Kelly 2011). Adaptation in No Bee populations caused a rapid increase in selfed seed production in the first five generations, yet subsequent phenotypic evolution plateaued (Table 1). Even though the reasons for this attenuated phenotypic response remain unknown, the loss of evolutionary potential in populations without pollinators presents a reasonable hypothesis to challenge in future work. The evolution of selfing has long been considered as a blind alley, where the loss of genetic variation precludes adaptive responses to changing environmental conditions (Stebbins 1957; Wright et al. 2013). Inferring the causes of limited genetic variation in selfers is challenging long after such declines have occurred (Glemin et al. 2006; Wang et al. 2021). In this short‐term experiment, the loss of genetic variation and its genome‐wide pattern are best explained by natural selection and a wide‐ranging influence of genetic draft in fully selfing lineages (Caballero and Santiago 1995; Gillespie 2001; Roze 2016). The degree to which the longevity of highly selfing lineages is explained by limits on recombination warrants broader investigation.

## AUTHOR CONTRIBUTIONS

SBR conducted the greenhouse experiment and ST prepared DNA libraries. JWB and JKK wrote programs to analyze sequence data. ST, JWB, and JKK wrote simulations. JWB developed an initial manuscript draft and all authors contributed to writing. JWB and JKK secured research funding.

## CONFLICT OF INTEREST

The authors declare no conflict of interest.

## DATA ARCHIVING

Sequence data are accessible from the NCBI SRA under PRJNA822425. Code is available on Dryad (https://doi.org/10.5061/dryad.h44j0zpnm).

Associate Editor: Y. Brandvain

Handling Editor: T. Chapman

## Supporting information

Table S1. Annotations for SNPs that reject the null model of genetic drift. Best model represents the implicated model of adaptation (Table 2). For non‐coding sites, the nearest gene was identified in M. guttatus. Orthologous genes in A. thaliana are based on highest sequence similarity to the M. guttatus gene. ‘NA’ denotes ‘not available,’ while ‘–’ denotes identity with annotations in the row immediately above.
Figure S1. The distributions of nucleotide diversity (pi) in No Bee (A1, A2) and Bee populations (B1, B2) along each chromosome. The number of pairwise differences per site was calculated within 50 kb windows on each chromosome.
Figure S2. The null variance produced in neutral simulations with full selfing. At a given population size, the number of individuals (N) was held constant across 9 generations of evolution. Outlines denote distributions and filled circles denote the mean over 1000 independent replicates.
Figure S3. The outcomes of SNP outlier tests. P‐value distributions were generated from likelihood ratio tests of adaptation in a single population (A1: model 1, A2: model 2, B1: model3, B2: model 4; Table 2). Red lines depict threshold P‐values for significant outliers with genome‐wide FDR = 0.05. Tests of adaptation in No Bee populations (A1, A2) fail to produce P‐values that approach the threshold significance level. Filled circles denote means.
