# Supplementary material for: Pollinator loss causes rapid adaptive evolution of selfing and dramatically reduces genome‐wide genetic variability
Source: Evolution. 2022 Jul 25;76(9):2130–44. doi: 10.1111/evo.14572 (PMC9543508; doi:10.1111/evo.14572)
Supplement: Supplementary file 1 — Table S1. Annotations for SNPs that reject the null model of genetic drift. Best model represents the implicated model of adaptation (Table 2). For non‐coding sites, the nearest gene was identified in M. guttatus. Orthologous genes in A. thaliana are based on highest sequence similarity to the M. guttatus gene. ‘NA’ denotes ‘not available,’ while ‘–’ denotes identity with annotations in the row immediately above. Figure S1. The distributions of nucleotide diversity (pi) in No Bee (A1, A2) and Bee populations (B1, B2) along each chromosome. The number of pairwise differences per site was calculated within 50 kb windows on each chromosome. Figure S2. The null variance produced in neutral simulations with full selfing. At a given population size, the number of individuals (N) was held constant across 9 generations of evolution. Outlines denote distributions and filled circles denote the mean over 1000 independent replicates. Figure S3. The outcomes of SNP outlier tests. P‐value distributions were generated from likelihood ratio tests of adaptation in a single population (A1: model 1, A2: model 2, B1: model3, B2: model 4; Table 2). Red lines depict threshold P‐values for significant outliers with genome‐wide FDR = 0.05. Tests of adaptation in No Bee populations (A1, A2) fail to produce P‐values that approach the threshold significance level. Filled circles denote means. [file EVO-76-2130-s001.docx]

**Supplementary Materials**

**Table S1.** Annotations for SNPs that reject the null model of genetic drift. Best model represents the implicated model of adaptation (Table 2). For non-coding sites, the nearest gene was identified in *M. guttatus*. Orthologous genes in *A. thaliana* are based on highest sequence similarity to the *M. guttatus* gene. ‘NA’ denotes ‘not available,’ while ‘--’ denotes identity with annotations in the row immediately above.

| **Chrom.** | **SNP position** | **Best model** | **LRT *P* value** | **Type of mutation** | ***Mimulus guttatus* gene** | ***A. thaliana* ortholog** | **Other names** | **Gene Ontology Biological Process** |
| --- | --- | --- | --- | --- | --- | --- | --- | --- |
| 1 | 2600671 | 8 | 3.81e-5 | non-coding | MgTOL.A0521 | AT5G07840 | Ankyrin repeat family protein | protein binding |
| 1 | 2600678 | 8 | 4.56e-5 | non-coding | -- | -- | -- | -- |
| 2 | 17560911 | 3 | 1.15e-9 | non-coding | MgTOL.B1848 | AT4G01037 | Ubiquitin carboxyl-terminal hydrolase family protein | NA |
| 2 | 17562471 | 3 | 9.22e-9 | non-coding | MgTOL.B1847 | NA | NA | DNA binding |
| 2 | 17643121 | 3 | 2.70e-7 | non-coding | MgTOL.B1864 | AT2G46494 | RING/U-box superfamily protein | protein binding; zinc ion binding |
| 2 | 17671461 | 3 | 2.21e-7 | non-coding | MgTOL.B1867 | AT5G42080 | dynamin-like protein | GTPase activity; GTP binding |
| 14 | 1368452 | 4 | 1.18e-7 | silent | MgTOL.N0274 | AT3G23300 | S-adenosyl-L-methionine-dependent methyltransferase | methyltransferase activity |
| 14 | 2034499 | 4 | 2.42e-7 | non-coding | MgTOL.N0401 | NA | NA | metabolic process; methyltransferase activity |
| 14 | 2376177 | 4 | 6.17e-8 | non-coding | MgTOL.N0457 | NA | NA | structural constituent of cell wall; plant-type cell wall organization |
| 14 | 2399466 | 4 | 7.28e-8 | non-coding | MgTOL.N0462 | AT2G43210 | Ubiquitin-like superfamily protein | protein binding |
| 14 | 2686014 | 4 | 2.00e-7 | non-coding | MgTOL.N0515 | AT4G20350 | oxidoreductases | oxidoreductase activity; |
| 14 | 2733738 | 4 | 1.62e-7 | non-coding | MgTOL.N0523 | AT4G26400 | RING/U-box superfamily protein | protein binding; zinc ion binding |
| 14 | 2742909 | 4 | 5.57e-9 | silent | MgTOL.N0525 | AT5G56360 | calmodulin-binding protein | structural molecule activity; protein binding; N-glycan processing; viral capsid |
| 14 | 2749877 | 4 | 4.41e-8 | non-coding | -- | -- | -- | -- |
| 14 | 2752619 | 4 | 8.94e-8 | non-coding | MgTOL.N0526 | NA | NA | structural constituent of ribosome; intracellular anatomical structure; ribosome; translation |
| 14 | 2752633 | 4 | 1.00e-7 | non-coding | -- | -- | -- | -- |
| 14 | 2872993 | 4 | 2.33e-7 | non-coding | MgTOL.N0544 | AT4G22380 | Ribosomal protein L7Ae/L30e/512e/Gadd45 family protein | RNA binding; structural constituent of ribosome; intracellular anatomical structure; nucleolus; ribosome; translation |
| 14 | 2873002 | 4 | 1.75e-7 | non-coding | -- | -- | -- | -- |
| 14 | 2873011 | 4 | 2.33e-7 | non-coding | -- | -- | -- | -- |
| 14 | 2873019 | 4 | 9.16e-7 | non-coding | -- | -- | -- | -- |
| 14 | 2873026 | 4 | 4.04e-8 | intron | MgTOL.N0545 | AT5G18120 | APR-like 7 | cell redox homeostasis |
| 14 | 2873027 | 4 | 3.61e-8 | intron | -- | -- | -- | -- |
| 14 | 2873057 | 4 | 8.58e-8 | silent | -- | -- | -- | -- |
| 14 | 2873123 | 4 | 3.01e-7 | silent | -- | -- | -- | -- |
| 14 | 2873160 | 4 | 9.62e-9 | intron | -- | -- | -- | -- |
| 14 | 2873191 | 4 | 6.94e-9 | non-coding | MgTOL.N0544 | AT4G22380 | Ribosomal protein L7Ae/L30e/512e/Gadd45 family protein | RNA binding; structural constituent of ribosome; intracellular anatomical structure; nucleolus; ribosome; translation |
| 14 | 2873192 | 4 | 5.78e-9 | non-coding | -- | -- | -- | -- |
| 14 | 2873197 | 4 | 6.22e-9 | non-coding | -- | -- | -- | -- |
| 14 | 2876891 | 4 | 4.39e-9 | non-coding | MgTOL.N0545 | AT5G18120 | APR-LIKE 7 | cell redox homeostasis |
| 14 | 2878354 | 4 | 7.42e-8 | non-coding | -- | -- | -- | -- |
| 14 | 2932325 | 4 | 9.52e-8 | intron | MgTOL.N0552 | NA | NA | nucleotide binding; nucleic acid binding; zinc ion binding |
| 14 | 2934927 | 4 | 1.28e-7 | non-coding | -- | -- | -- | -- |
| 14 | 2956648 | 4 | 2.49e-7 | non-coding | MgTOL.N0555 | AT5G01430 | Got1/Sft2-like vesicle transport protein family | vesicle-mediated transport |
| 14 | 2956654 | 4 | 2.07e-7 | non-coding | -- | -- | -- | -- |
| 14 | 2956656 | 4 | 6.81e-8 | non-coding | -- | -- | -- | -- |
| 14 | 2956686 | 4 | 5.45e-8 | non-coding | -- | -- | -- | -- |
| 14 | 2956697 | 4 | 1.10e-7 | non-coding | -- | -- | -- | -- |
| 14 | 2956704 | 4 | 8.39e-8 | non-coding | -- | -- | -- | -- |
| 14 | 2956712 | 4 | 8.41e-8 | non-coding | -- | -- | -- | -- |
| 14 | 2956746 | 4 | 1.86e-8 | non-coding | -- | -- | -- | -- |
| 14 | 3376570 | 4 | 2.17e-8 | non-coding | MgTOL.N0625 | AT3G60880 | dihydrodipicolinate synthase 1 | catalytic activity; metabolic process; 4-hydroxy-tetrahydrodipicilinate synthase activity; lysine biosynthetic process via diaminopimelate; lyase activity |
| 14 | 3605475 | 4 | 2.21e-7 | non-coding | MgTOL.N0650 | AT5G12230 | lung-cancer metastasis-related LCMR1 protein | NA |
| 14 | 2605479 | 4 | 1.52e-7 | non-coding | -- | -- | -- | -- |
| 14 | 3605483 | 4 | 1.42e-7 | non-coding | -- | -- | -- | -- |
| 14 | 3605485 | 4 | 2.33e-7 | non-coding | -- | -- | -- | -- |
| 14 | 3605487 | 4 | 1.44e-7 | non-coding | -- | -- | -- | -- |
| 14 | 3613830 | 4 | 2.85e-8 | non-coding | -- | -- | -- | -- |


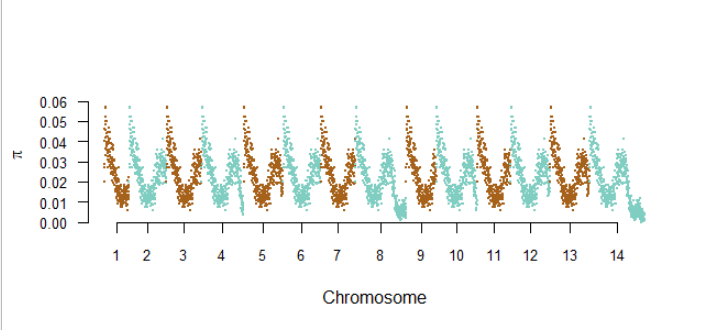

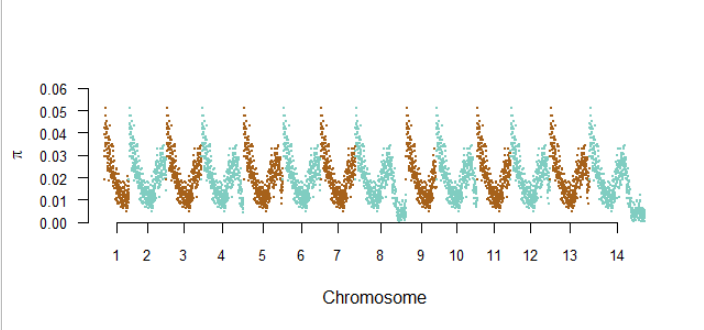

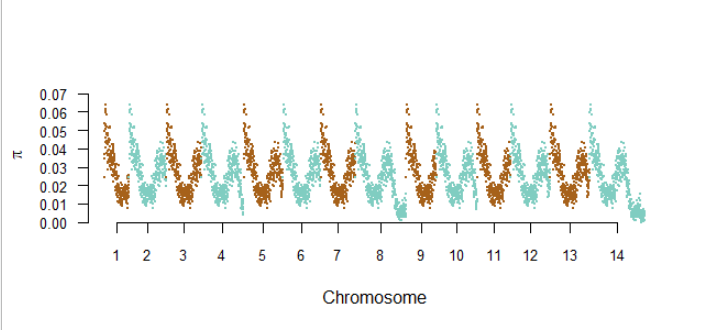

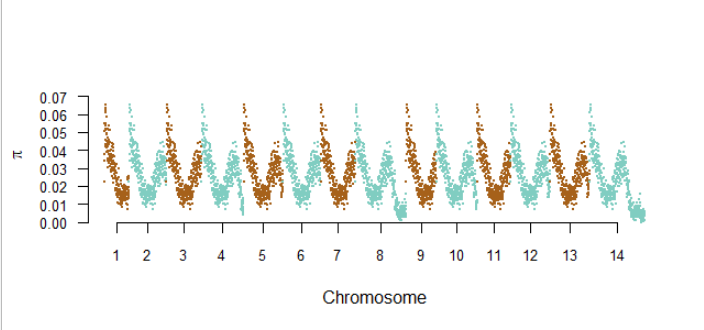


**A1**

**A2**

**B1**

**B2**

**Figure S1.** The distributions of nucleotide diversity (π) in No Bee (A1, A2) and Bee populations (B1, B2) along each chromosome. The number of pairwise differences per site was calculated within 50 kb windows on each chromosome.


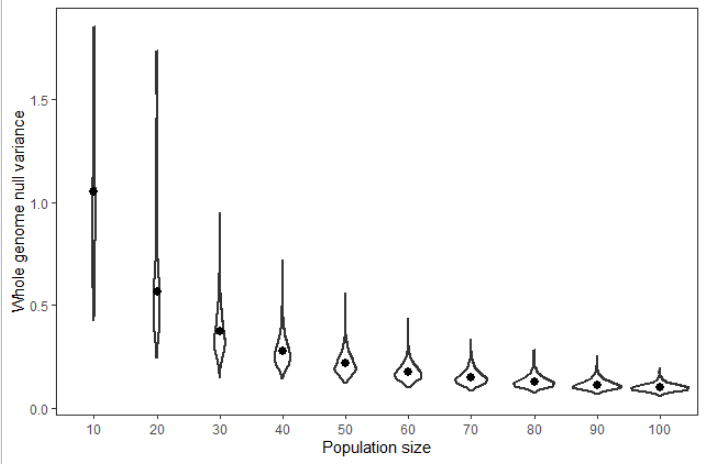


**Figure S2.** The null variance produced in neutral simulations with full selfing. At a given population size, the number of individuals (*N*) was held constant across 9 generations of evolution. Outlines denote distributions and filled circles denote the mean over 1000 independent replicates.


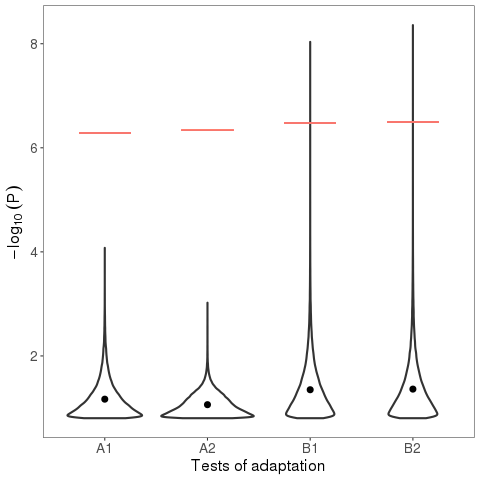


**Figure S3.**  The outcomes of SNP outlier tests. P-value distributions were generated from likelihood ratio tests of adaptation in a single population (A1: model 1, A2: model 2, B1: model3, B2: model 4; Table 2). Red lines depict threshold P-values for significant outliers with genome-wide FDR = 0.05. Tests of adaptation in No Bee populations (A1, A2) fail to produce P-values that approach the threshold significance level. Filled circles denote means.
